# Computer-Assisted Orthopedic Surgery: Current State and Future Perspective

**DOI:** 10.3389/fsurg.2015.00066

**Published:** 2015-12-23

**Authors:** Guoyan Zheng, Lutz P. Nolte

**Affiliations:** ^1^Institute for Surgical Technology and Biomechanics, University of Bern, Bern, Switzerland

**Keywords:** computer-assisted orthopedic surgery, surgical navigation, medical robotics, orthopedics, imaging, registration, referencing

## Abstract

Introduced about two decades ago, computer-assisted orthopedic surgery (CAOS) has emerged as a new and independent area, due to the importance of treatment of musculoskeletal diseases in orthopedics and traumatology, increasing availability of different imaging modalities, and advances in analytics and navigation tools. The aim of this paper is to present the basic elements of CAOS devices and to review state-of-the-art examples of different imaging modalities used to create the virtual representations, of different position tracking devices for navigation systems, of different surgical robots, of different methods for registration and referencing, and of CAOS modules that have been realized for different surgical procedures. Future perspectives will also be outlined.

## 1 Introduction

The human musculoskeletal system is an organ system that includes the bones of the skeleton and the cartilages, ligaments, and other connective tissues that bind tissues and organs together. The main functions of this system are to provide form, support, stability, and movement to the body. Bones, besides supporting the weight of the body, work together with muscles to maintain body position and to produce controlled, precise movements. Musculoskeletal disease is among the most common causes of severe long-term disability and practical pain in industrialized societies (1). The impact and importance of musculoskeletal diseases are critical not only for individual health and mobility but also for social functioning and productivity and economic growth on a larger scale, reflected by the proclamation of the Bone and Join Decade 2000–2010 (1).

Both traumatology and orthopedic surgery aim at the treatment of musculoskeletal tissues. Surgical steps, such as the placement of an implant component, the reduction and fixation of a fracture, ligament reconstruction, osteotomy, tumor resection, and the cutting or drilling of bone, should ideally be carried out as precisely as possible. Not only will optimal precision improve the post-operative outcome of the treatment, but it will also minimize the risk factors for intra- and post-operative complications. To this end, a large number of pure mechanical guides have been developed for various clinical applications. The pure mechanical guides, though easy to use and easy to handle, does not respect the individual patient’s morphology. Thus, their general benefit has been questioned [see, for example, Ref. (2)]. Additionally, surgeons often encounter the challenge of limited visibility of the surgical situs, which makes it difficult to achieve the intended procedure as accurately as desired. Moreover, the recent trend toward increased minimally invasive surgery makes it more and more important to gain feedback about surgical actions that take place subcutaneously. Just as a global position system (GPS)-based car navigation provides visual instruction to a driver by displaying the location of the car on a map, a computer-assisted orthopedic surgery (CAOS) module allows the surgeon to get real-time feedback about the performed surgical actions using information conveyed through a virtual scene of the situs presented on a display device (3, 4). Parallel to the CAOS module to potentially improve surgical outcome is the employment of surgical robots that actively or semi-actively participate in the surgery (5).

Introduced about two decades ago (3–5), CAOS has emerged as a new and independent area and stands for approaches that use computer-enabled tracking systems or robotic devices to improve visibility to the surgical field and increase application accuracy in a variety of surgical procedures. Although CAOS modules use numerous technical methods to realize individual aspects of a procedure, their basic conceptual design is very similar. They all involve three major components: a therapeutic object [(TO), which is the target of the treatment], a virtual object [(VO), which is the virtual representation in the planning and navigation computer], and a so-called navigator that links both objects. For reasons of simplicity, the term “CAOS system” will be used within this article to refer to both navigation systems and robotic devices.

The central element of each CAOS system is the navigator. It is a device that establishes a global, three-dimensional (3-D) coordinate system (COS) in which the target is to be treated and the current location and orientation of the utilized end-effectors (EEs) are mathematically described. EEs are usually passive surgical instruments, but can also be semi-active or active devices. One of the main functions of the navigator is to enable the transmission of positional information between the EEs, the TO, and the VO. For robotic devices, the robot itself plays the role of the navigator; while for surgical navigation, a position tracking device is used.

For the purpose of establishment of a CAOS system through co-actions of these three entities, three key procedural requirements have to be fulfilled. The first is the calibration of the EEs, which means to describe the EEs’ geometry and shape in the COS of the navigator. For this purpose, it is required to establish physically a local COS at the EEs. When an optical tracker is used, this is done via rigid attachment of three or more optical markers onto each EE. The second is registration, which aims to provide a geometrical transformation between the TO and the VO in order to display the end-effect’s localization with respect to the virtual representation, just like display of the location of a car in a map in a GPS-based navigation system. The geometrical transformation could be rigid or non-rigid. In the literature, a wide variety of registration concepts and associated algorithms exist (see the next section for more details). The third key ingredient to a CAOS system is referencing, which is necessary to compensate for possible motion of the navigator and/or the TO during the surgical actions to be controlled. This is done by either attaching a so-called “dynamic reference bases (DRB)” holding three or more optical markers to the TO or immobilizing the TO with respect to the navigator.

The rest of the paper is organized as follows. Section 2 will review the state-of-the-art examples of basic elements of CAOS systems. Section 3 will present clinical fields of applications. In Section 4, future perspectives will be outlined, followed by conclusion in Section 5.

## 2 Basic Elements of CAOS Systems

### 2.1 Virtual Object

**Figure 1:**
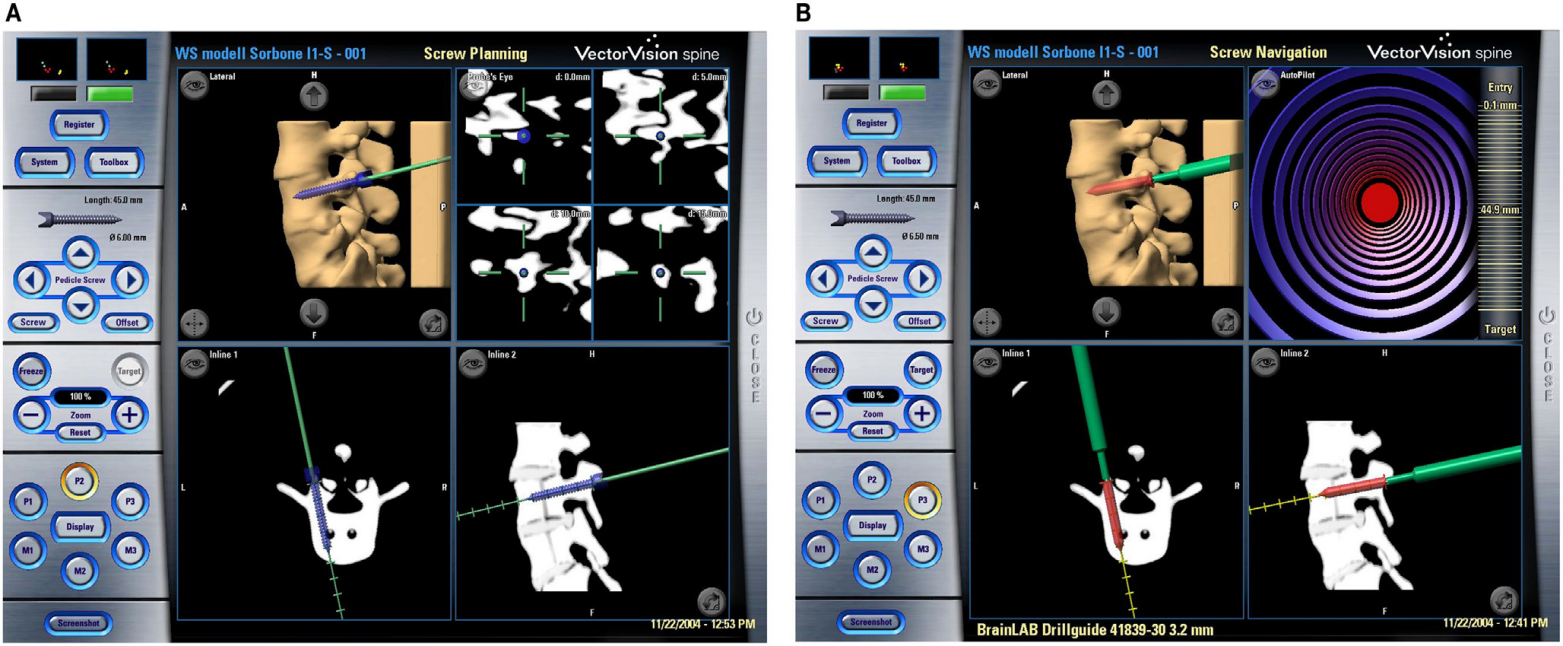
**Example of CT-based navigational feedback**. These screenshots show a CT-based CAOS system during pre-operative planning **(A)** and intra-operative navigation **(B)** of pedicle screw placement (Courtesy of BrainLAB AG, Munich, Germany).

The VO in each CAOS system is defined as a sufficiently realistic representation of the musculoskeletal structures that allow the surgeon to plan the intended intervention, as exemplified in Figure 1A. Intra-operatively, it also serves as the “back-ground” into which the measured position of a surgical instrument can be visualized (see Figure 1B for an example). Though most of time VO is derived from image data of the patient, it can also be created directly from intra-operative digitization without using any medical image data. Below, detailed examples of different forms of VOs will be reviewed.

**Figure 2:**
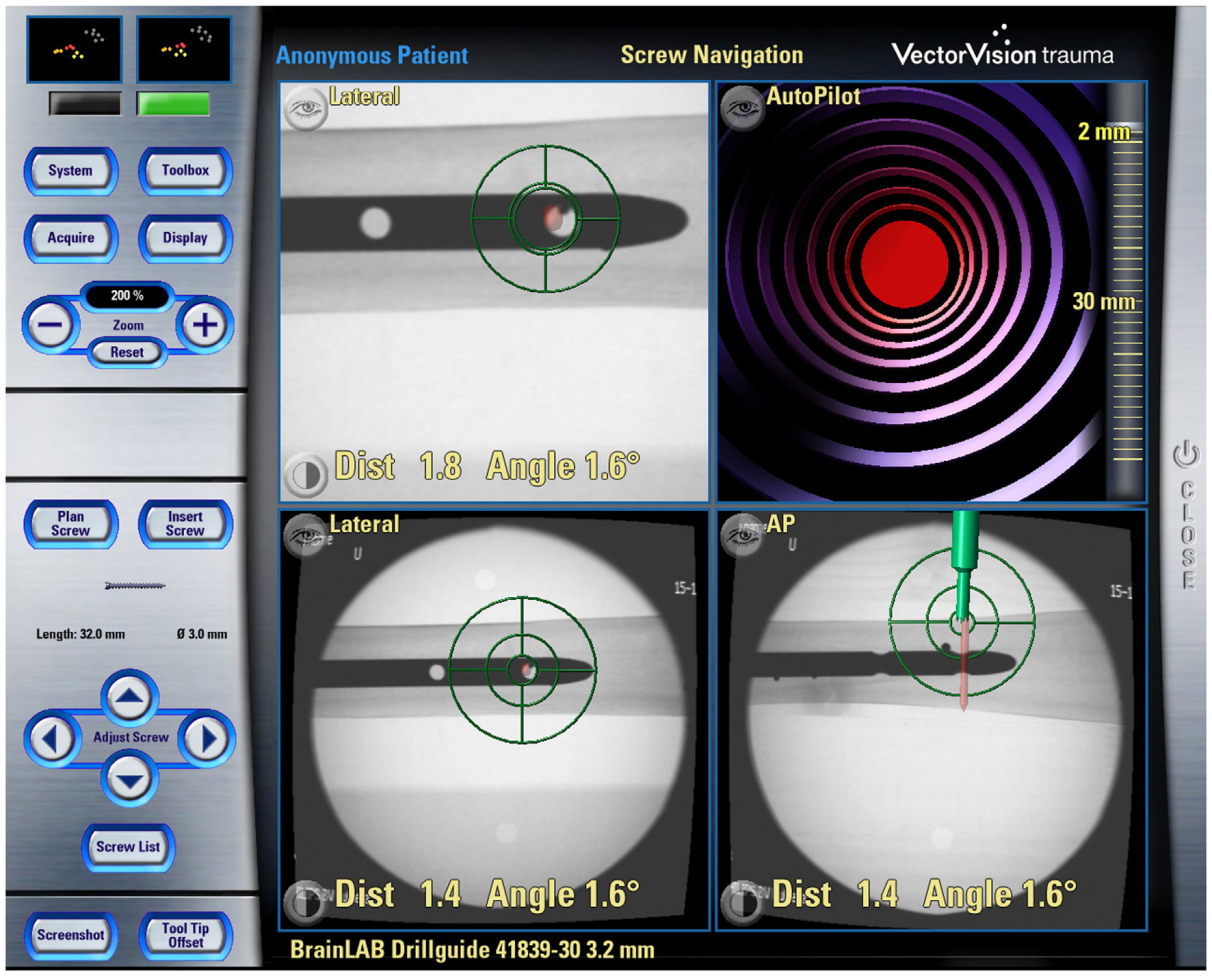
**Example of Fluoroscopy-based navigation**. This screenshot shows the fluoroscopy-based navigation for distal locking of an intramedullary nail (Courtesy of BrainLAB AG, Munich, Germany).

When the VO is derived from medical image data, these data may be acquired at two points in time: either pre-operatively or intra-operatively. About two decades ago, the VOs of majority CAOS systems were derived from pre-operatively acquired CT scans, and a few groups also tried to use magnetic resonance imaging (MRI) (6, 7). In comparison with MRI, CT has clear advantages of excellent bone–soft tissue contrast and no geometrical distortion despite its acquisition inducing radiation exposure to the patient. Soon after the introduction of the first CAOS systems, the limitations of pre-operative VOs were observed, which led to the introduction of intra-operative imaging modalities. More specifically, the bony morphology may have changed between the time of image acquisition and the actual surgical procedure. As a consequence, the VO may not necessarily correspond to the TO any more leading to unpredictable inaccuracies during navigation or robotic procedures. This effect can be particularly adverse for traumatology in the presence of unstable fractures. To overcome this problem in the field of surgical navigation, the use of intra-operative CT scanning has been proposed (8), but the infrastructural changes that are required for the realization of this approach are tremendous, often requiring considerable reconstruction of a hospital’s facilities. This has motivated the development of navigation systems based on fluoroscopic images (9–11). The image intensifier is a well-established device during orthopedic and trauma procedures but has the limitations that the images generated with a fluoroscope is usually distorted and that one-dimensional information gets lost due to image projection. To use these images as VOs, therefore, requires the calibration of the fluoroscope that aims to compute the image projection model and to compensate for the image distortion (9–11). The resultant systems are, therefore, known as “fluoroscopy-based navigation systems” in the literature (9–11). Additional feature offered by a fluoroscopy-based navigation system is that multiple images acquired from different positions are co-registered to a common COS established on the target structure via the DRB technique. Such a system can, thus, provide visual feedback just like the use of multiple fluoroscopes placed at different positions in constant mode but without the associated radiation exposure (see Figure 2 for an example), which is a clear advantage. This technique is, therefore, also known as “virtual fluoroscopy” (11). Despite the fact that in such a system only two-dimensional (2-D) projected images with low contrast are available, the advantages offered by a fluoroscopy-based navigation system preponderate for a number of clinical applications in orthopedics and traumatology.

In order to address the 2-D projection limitation of a fluoroscopy-based navigation system, a new imaging device was introduced (12) that enables the intra-operative generation of 3-D fluoroscopic image data. It consists of a motorized, iso-centric C-arm that acquires series of 50–100, 2-D projections and reconstructs from them 13 cm × 13 cm × 13 cm volumetric datasets that are comparable to CT scans. Being initially advocated primarily for surgery at the extremities, this “fluoro-CT” has been adopted for usage with a navigation system and has been applied to several anatomical areas already (13, 14). As a major advantage, the device combines the availability of 3-D imaging with the intra-operative data acquisition. “Fluoro-CT” technology is under continuous development involving smaller and non-iso-centric C-arms, “closed” C-arm, i.e., O-arm™ design (15, 16), faster acquisition speeds, larger field of view, and also flat panel technology.

**Figure 3:**
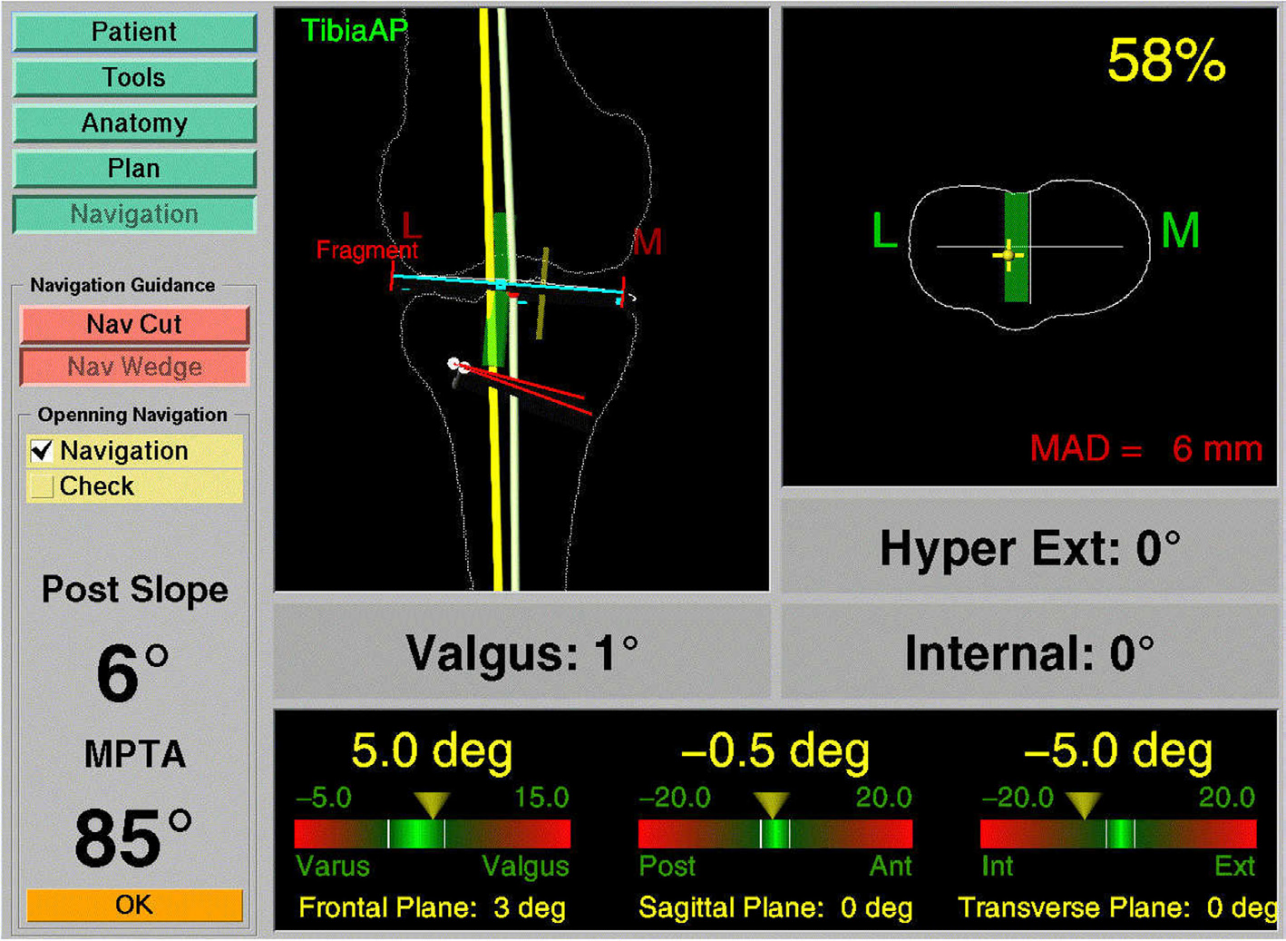
**Navigation using surgeon-defined anatomy approach**. This virtual model of a patient’s knee is generated intra-operatively by digitizing relevant structures. Although a very abstract representation, it provides sufficient information to enable navigated high tibial osteotomy.

**Figure 4:**
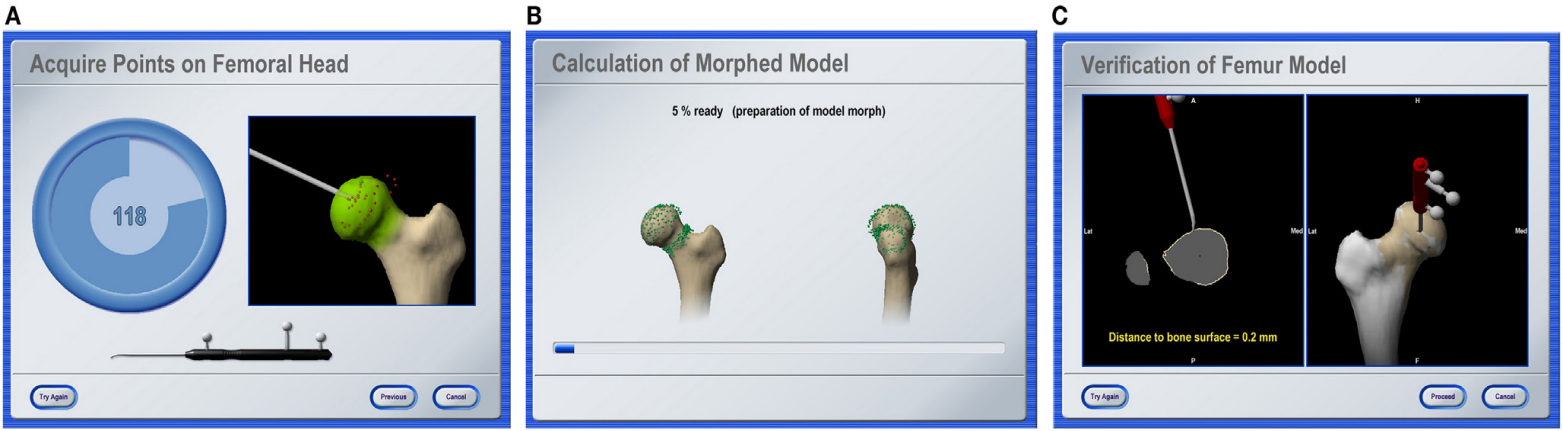
**Bone morphing**. Screenshots of different stages of an intra-operative bone morphing process. **(A)** Point acquisition; **(B)** calculation of morphed model; and **(C)** verification of final result (Courtesy of BrainLAB AG, Munich, Germany).

A last category of navigation systems functions without any radiological images as VOs. Instead, the tracking capabilities of the system are used to acquire a graphical representation of the patient’s anatomy by intra-operative digitization. By sliding the tip of a tracked instrument on the surface of a surgical object, the spatial location of points on the surface can be recorded. Surfaces can then be generated from the recorded sparse point clouds and used as the virtual representation of the surgical object. Because this model is generated by the operator, the technique is, therefore, known as “surgeon-defined anatomy” (SDA). It is particularly useful when soft tissue structures, such as ligaments or cartilage boundaries, are to be considered that are difficult to identify on CTs or fluoroscopic images (17). Moreover, with SDA-based systems some landmarks can be acquired even without the direct access to the anatomy. For instance, the center of the femoral head, which is an important landmark during total hip and knee replacement, can be calculated from a recorded passive rotation of the leg about the acetabulum. It should be noted that the generated representations are often rather abstract and not easy to interpret as exemplified in Figure 3. This has motivated the developed of the so-called “bone morphing” techniques (18, 19), which aim to derive a patient-specific model from a generic statistical forms of the target anatomical structure and a set of sparse points that are acquired with the SDA technique (20). As a result, a realistic virtual model of the target structure can be presented and used as a VO without any conventional image acquisition (Figure 4).

### 2.2 Registration

Position data that are used intra-operatively to display the current tool location (navigation system) or to perform automated actions according to a pre-operative plan (robot) are expressed in the local COS of the VO. In general, this COS differs from the one in which the navigator operates intra-operatively. In order to bridge this gap, the mathematical relationships between both coordinate spaces needs to be determined. When pre-operative images are used as VOs, this step is performed interactively by the surgeon during the registration, also known as matching. A wide variety of different approaches have been developed and realized following numerous methodologies (21).

Early CAOS systems implemented paired-point matching and surface matching (22). The operational procedure for paired-point matching is simple. Pairs of distinct points are defined pre-operatively in the VO and intra-operatively in the TO. The points on the VO are usually identified pre-operatively using the computer mouse, while the corresponding points on the TO are usually done intra-operatively with a tracked probe. In the case of a navigation system, the probe is tracked by the navigator; and for a robotic surgery, it is mounted onto the robot’s actuator (23). Although paired-point matching is easy to solve mathematically, the accuracy of the resultant registration is low. This is due to the fact that the accuracy of paired-point matching depends on an optimal selection of the registration points and the exact identification of the associated pairs which is error-prone. One obvious solution to this problem is to implant artificial objects to create easily and exactly identifiable fiducials for an accurate paired-point matching (23). However, the requirement of implanting these objects before the intervention causes extra operation as well as associated discomfort and infection risk for the patient (24). Consequently, none of these methods have gained wide clinical acceptance. The other alternative that has been widely adopted in early CAOS systems is to complement the paired-point matching with surface matching (25, 26), which does not require implanting any artificial object and only uses the surfaces of the VO as a basis for registration.

**Figure 5:**
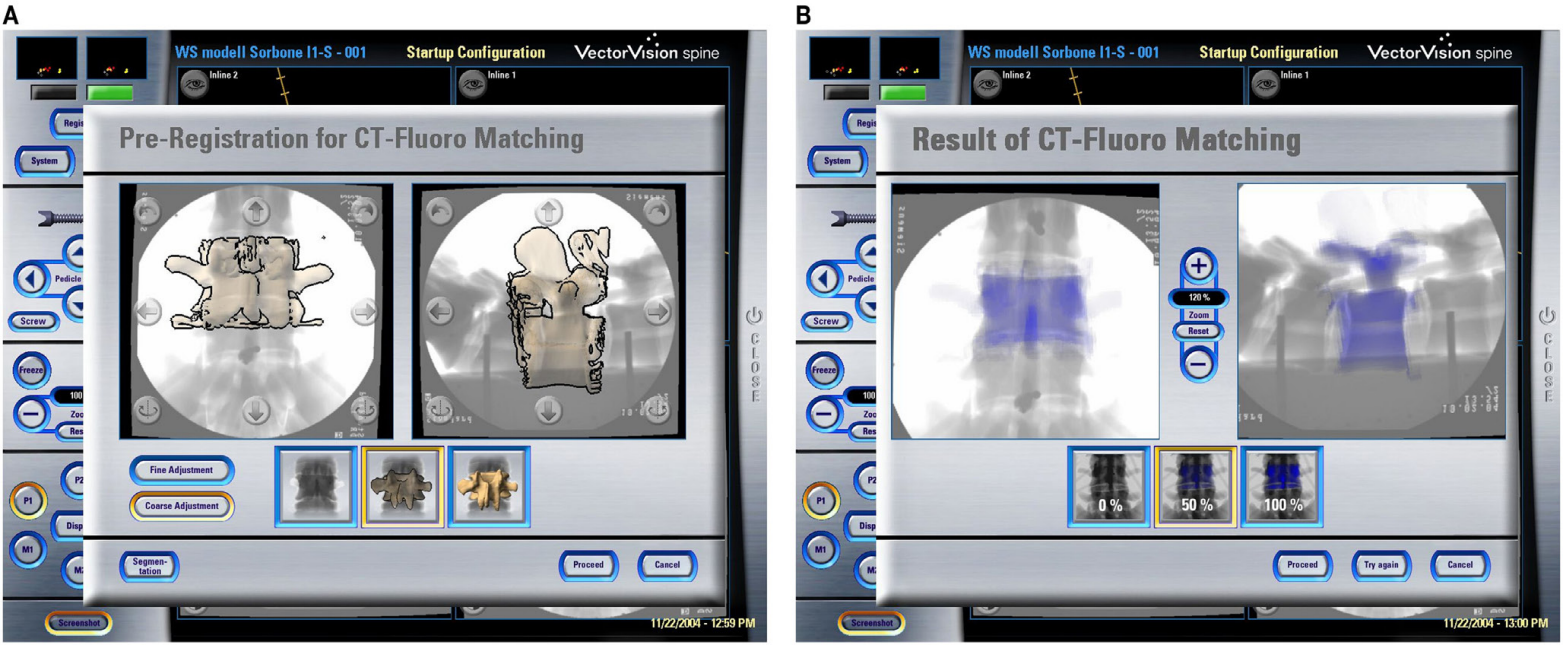
**CT-Fluoro matching**. Screenshots of different stages of a CT-Fluoro matching process. **(A)** Pre-registration for CT-Fluoro matching; and **(B)** results of CT-Fluoro matching (Courtesy of BrainLAB AG, Munich, Germany).

Other methods to compute the registration transformation without the need for extensive pre-operative preparation utilize intra-operative imaging, such as calibrated fluoroscopic images or calibrated ultrasound images. As described above, a limited number of fluoroscopic images (e.g., 2 images) acquired at different positions are calibrated and co-registered to a common COS established on the target structure. A so-called “2-D–3-D registration” procedure can then be used to find the geometrical transformation between the common COS and a pre-operatively acquired 3-D CT dataset by maximizing a similarity measurement between the 2-D projective representations and the associated digitally reconstructed radiographs (DRRs) that are created by simulating X-ray projections (see Figures 5A,B). Intensity-based as well as feature-based approaches have been proposed before. For a comprehensive review of different 2-D–3-D registration techniques, we refer to Ref. (21).

Another alternative is the employment of intra-operative ultrasonography. If an ultrasound probe is tracked by a navigator and its measurements are calibrated, it may serve as a spatial digitizer with which points or landmarks on the surfaces of certain subcutaneous bony structures may be acquired. This is different from the touch-based digitization done with a conventional probe that usually requires an invasive exposure of the surfaces of the target structures. Two different tracked mode ultrasound probes are available. A-(amplitude-) mode ultrasound probes can measure the depth along the acoustic axis of the device. Placed on the patient’s skin, they can measure percutaneously the distance to tissue borders, and the resulting point coordinates can be used as inputs to any feature-based registration algorithm. The applicability of this technique has been demonstrated previously but with certain limitations that prevent its wide usage (27, 28). More specifically, the accuracy of the A-mode ultrasound probe-based digitization depends on how well the probe can be placed perpendicularly to the surfaces of the target bony structures, which is not an easy task when the subcutaneous soft tissues are thick. Moreover, the velocity of sound during the probe calibration is usually different from the velocity of sound when the probe is used for digitization as the latter depends on the properties of the traversed tissues. Such a velocity difference will lead to unpredictable inaccuracies when the probe is used to digitize deeply located structures. As a consequence, the successful application of this technique remains limited to a narrow field of application. In contrast to an A-mode probe, a B-(brightness-) mode ultrasound probe scans a fan-shaped area. It is, therefore, able to detect also surfaces that are examined from an oblique direction, though the errors caused by the velocity difference still persist. In order to extract the relevant information for the registration of pre-operative CT scans, the resulting, usually noisy images need to be processed (29). As for the intra-operative processing of fluoroscopic images, the use of B-mode ultrasound for registration is not reliable in every case and consequently remains subject of CAOS research (30, 31).

It is worth to point out that if the VO is generated intra-operatively, registration is an inherent process (21). This is due to the fact that since the imaging device is tracked during data acquisition, the position of any acquired image is recorded with respect to the local COS established on the TO. The recorded device position, together with the additional image calibration process, automatically establishes the spatial relationship between the VO and the TO during image acquisition, which is a clear advantage over the interactive registration in the case of pre-operative images servings as VOs. Therefore, registration is not an issue when using intra-operative CT, 2-D, 3-D fluoroscopy or O-arm, or the SDA concept.

Radermacher et al. (32) introduced an alternative way to match pre-operative planning with the intra-operative situation using individual templates. The principle of individualized templates is to create customized templates based on patient-specific 3-D bone models that are normally segmented from pre-operative 3-D data, such as CT or MRI scan. One feature about the individual templates is that small reference areas of the bone structures are integrated into the templates as the contact faces. By this means, the planned position and orientation of the template in spatial relation to the bone are stored in a structural way and can be reproduced intra-operatively by adjusting the contact faces of the templates until an exact fit to the bone is achieved. By integrating holes and/or slots, individualized templates function as tool guides, e.g., for the preparation of pedicle screw holes (32) or as cutting jigs used in total knee and hip replacement surgery (33–35).

### 2.3 Navigator

Registration closes the gap between VO and TO. The navigator enables this connection by providing a global coordinate space. In addition, it links the surgical EEs, with which a procedure is carried out, to the TO that they act upon. From a theoretical standpoint, it is the only element in which surgical navigation systems and surgical robotic systems differ.

#### 2.3.1 Robots

For this type of CAOS technology, the robot itself is the navigator. Intra-operatively, it has to be registered to the VO in order to realize the plan that is defined in the pre-operative image dataset. The EEs of a robot are usually designed to carry out specific tasks as part of the therapeutic treatment. Depending on how the EEs of a robot act on the patient, two different types of robots can be found in the literature. The so-called active robots conduct a specific task autonomously without additional support by the surgeon. Such a system has been applied for total joint replacement (5) but their clinical benefit has been strongly questioned (36). For traumatology applications, the use of active robots has only been explored in the laboratory setting (37, 38). One possible explanation is that the nature of fracture treatment is an individualized process that does not include many steps that an active robot can repetitively carry out.

In contrast to active robotic devices, passive or semi-active robots do not carry out a part of the intervention autonomously, but rather guide or assist the surgeon in positioning the surgical tools. At present, there are two representatives of this class, both for bone resection during total knee replacement. The Navio system (Blue Belt Technologies Inc., Pittsburgh, PA, USA) (39) is a hand-held semi-active robotic technology for bone shaping that allows a surgeon to move freely in order to resect bone as long as this motion stays within a pre-operatively defined safety volume. The MAKO system (40) is a passive robotic-arm system providing oriental and tactile guidance. Both the Navio and the MAKO systems require additional tracking technology as described in the next sub-section. During the surgical procedure, the system is under the direct surgeon control and gives real-time tactile feedback to the surgeon. Other semi-active robots, such as SpineAssist (Mazor Robotics Ltd., Israel) can be seen as intelligent gages that place, e.g., cutting jigs or drilling guides automatically (41, 42).

#### 2.3.2 Trackers

The navigator of a surgical navigation system is a spatial position tracking device. It determines the location and orientation of objects and provides these data as 3-D coordinates or 3-D rigid transformations. Although a number of tracking methods based on various physical media, e.g., acoustic, magnetic, optical, and mechanical methods, have been used in the early surgical navigation systems, most of today’s products rely upon optical tracking of objects using operating room (OR) compatible infrared light that is either actively emitted or passively reflected from the tracked objects. To track surgical EEs with this technology then requires the tools to be adapted with reference bases holding either light emitting diodes (LED, active) or light reflecting spheres or plates (passive). Tracking patterns with known geometry by means of video images has been suggested (43, 44) as an inexpensive alternative to an infrared-light optical tracker.

Optical tracking of surgical EEs requires a direct line of sight between the tracker and the observed objects. This can be a critical issue in the OR setting. The use of electromagnetic tracking systems have been proposed to overcome this problem. This technology involves a homogeneous magnetic field generator that is usually placed near to the surgical situs and the attachment of receiver coils to each of the instruments allowing measuring their position and orientation within the magnetic field. This technique senses positions even if objects such as the surgeon’s hand are in between the emitter coil and the tracked instrument. However, the homogeneity of the magnetic field can be easily disturbed by the presence of certain metallic objects causing measurement artifacts that may decrease the achievable accuracy considerably (45–47). Therefore, magnetic tracking has been employed only in very few commercial navigation systems and with limited success.

Recently inertial measurement unit (IMU)-based navigation devices have attracted more and more interests (48–51). These devices attempt to combine the accuracy of large-console CAOS systems with the familiarity of conventional alignment methods and have been successfully applied to applications, including TKA (48, 49), pedicle screw placement (50), and periacetabular osteotomy (PAO) surgery (51). With such devices, the line-of-sight issues in the optical surgical navigation systems can be completely eliminated. Technical limitations of such devices include (a) relatively lower accuracy in comparison with optical tracking technique and (b) difficulty in measuring translations.

### 2.4 Referencing

**Figure 6:**
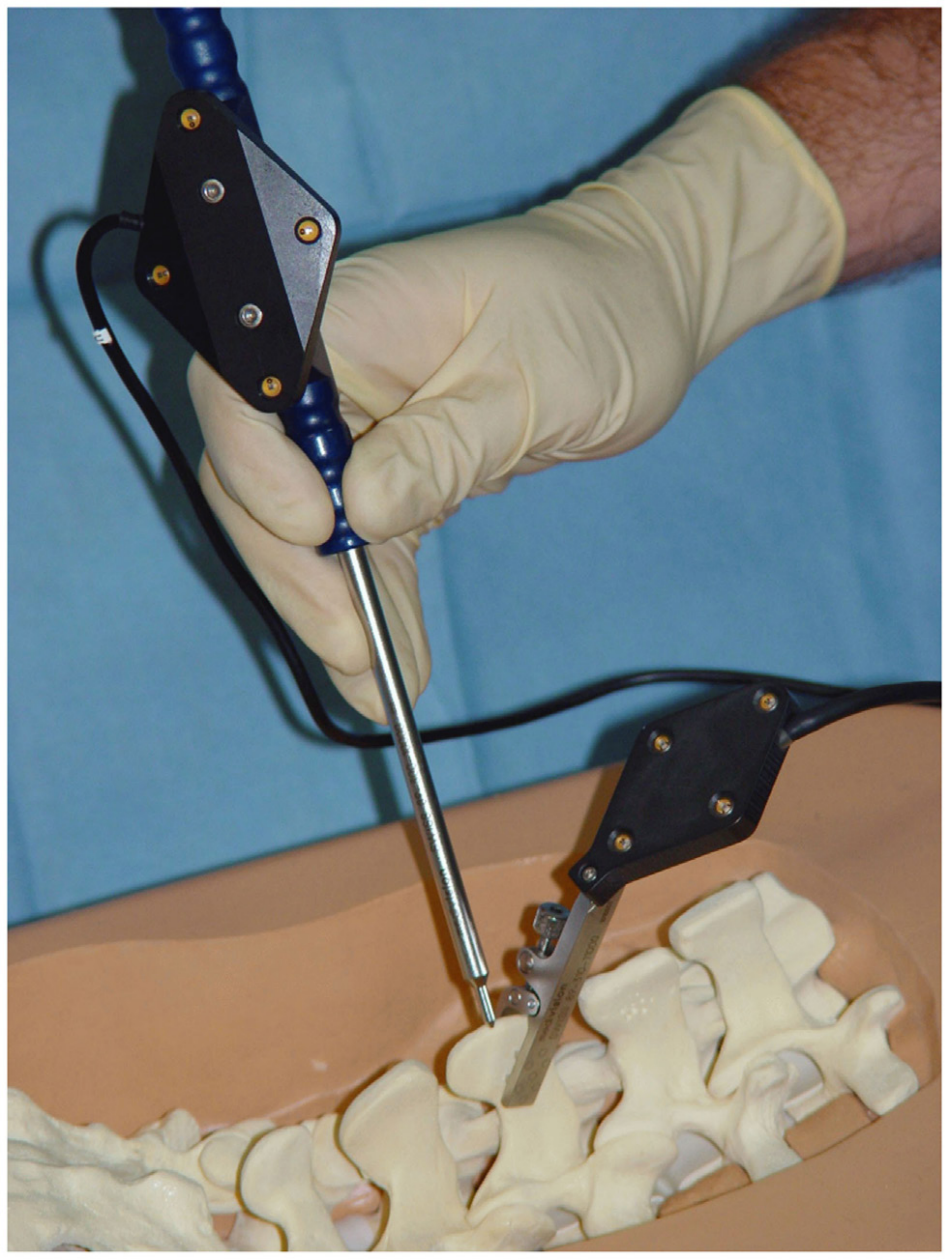
**Dynamic reference base**. A dynamic reference base allows a navigation system to track the anatomical structure that the surgeon is operating on. In the case of spinal surgery, this DRB is usually attached to the processus spinosus with the help of a clamping mechanism. It is essential that it remains rigidly affixed during the entire usage of the navigation system on that vertebra.

Intra-operatively, it is unavoidable that there will be relative motions between the TO and the navigator due to surgical actions. Such motions need to be detected and compensated to secure surgical precision. For this purpose, the operated anatomy is linked to the navigator. For robotic surgery, this connection is established as a physical linkage. Large active robots, such as the early machines used for total joint replacement, come with a bone clamp that tightly grips the treated structure or involve an additional multi-link arm, while smaller active and semi-active devices are mounted directly onto the bone. For all other tracker types, bone motion is determined by the attachment of a DRB to the TO (52), which is designed to house infrared LEDs, reflecting markers, acoustic sensors, or electromagnetic coils, depending on the employed tracking technology. Figure 6 shows an example of a DRB for an active optical tracking system that is attached to the spinous process of a lumbar vertebra. Since the DRB is used as an indicator to inform the tracker precisely about movements of the operated bone, a stable fixation throughout the entire duration of the procedure is essential.

## 3 Clinical Fields of Application

Since the mid-1990s when first CAOS systems were successfully utilized for the insertion of pedicle screws at the lumbar and thoracic spine and total hip replacement procedures (3, 4), a large number of modules covering a wide range of traumatological and orthopedic applications have been developed, validated in the laboratory and in clinical trials. Some of them needed to be abandoned, because the anticipated benefit failed to be achieved or the technology proved to be unreliable or too complex to be used intra-operatively. Discussing all these applications would go beyond the focus of this article. Thus, here we focus on a review of the most important applications with the most original technological approaches.

While there was clearly one pioneering example of robot-assisted orthopedic surgery – ROBODOC (5), the first spinal navigation systems were realized independently by several research groups, almost in parallel (3, 4, 52–56). These systems used pre-operative CT scans as the VO, relied upon paired-points and surface matching techniques for registration, and were based on optical or electromagnetic trackers. Their initial clinical success (57–59) boosted the development of new CAOS systems and modules. While some groups tried to use the existing pedicle screw placement systems for other clinical applications, others aimed to apply the underlying technical principle to new clinical challenges by developing highly specialized navigation systems (60, 61). With the advent of alternative imaging methods for the generation of VOs, the indication for the use of one or the other method was evaluated more critically. For instance, it became evident that lumbar pedicle screw insertion in the standard degenerative case could be carried out with fluoroscopy-based navigation sufficiently accurate; thus, avoiding the need for a pre-operative CT.

A similar development took place for total knee replacement. Initially, this procedure was supported by active (36, 62) and semi-active or passive (39, 40) robots, as well as navigation systems using pre-operative CTs (63) but with a few exceptions the SDA approach (64) is today’s method of choice.

Fluoroscopy-based navigation still seems to have a large potential to explore new fields of application. The technology has been mainly used in spinal surgery (65). Efforts to apply it to total hip replacement (66) and the treatment of long bone fractures (67) have been commercially less successful. The intra-operative 3-D fluoroscopy or O-arm has been explored intensively (13–16). It is expected that with the advent of the flat panel technology, the use of fluoro-CT as a virtual object generator will significantly grow (16).

**Figure 7:**
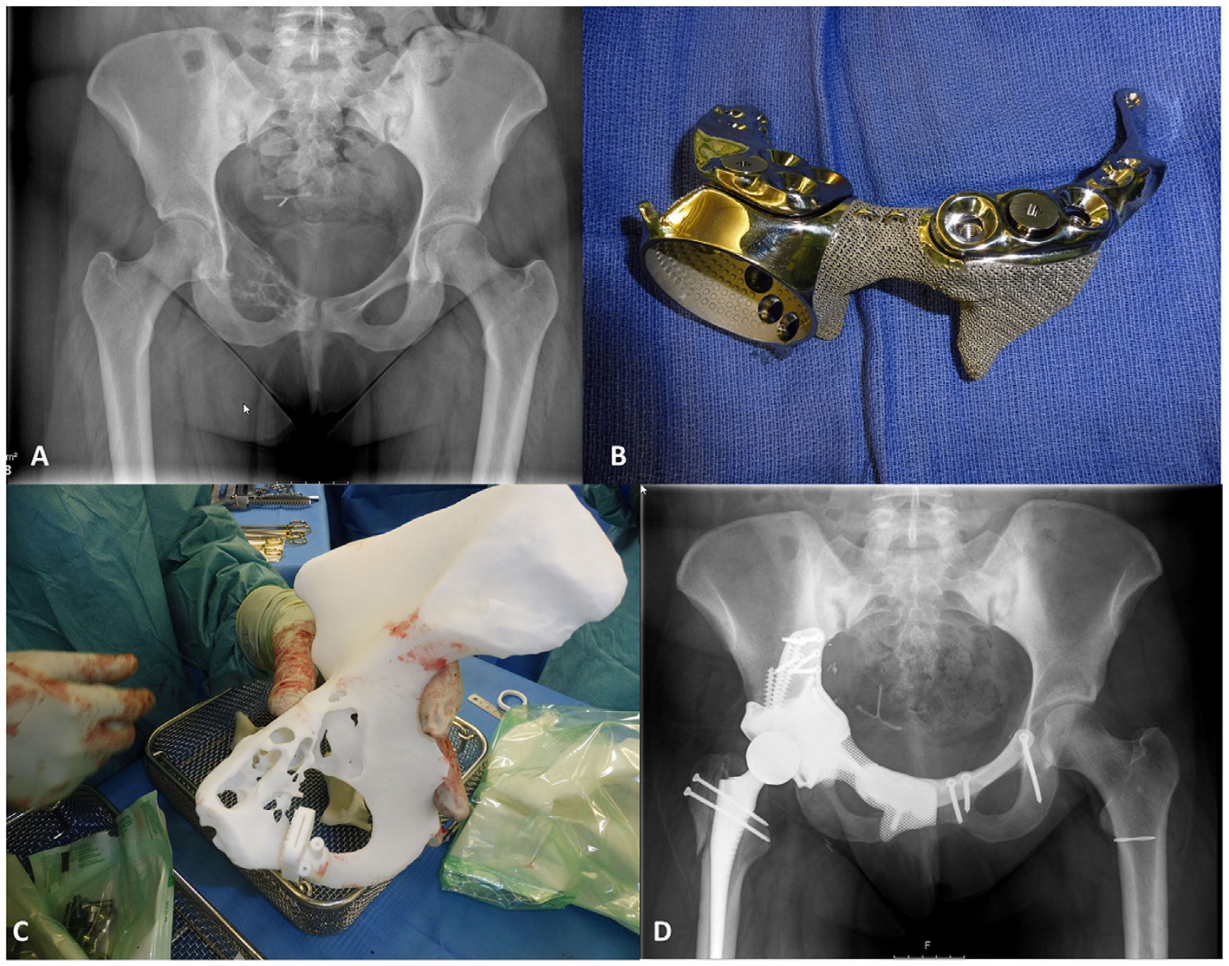
**Patient-specific instrumentation for pelvic tumor resection surgery**. These images show the application of patient-specific instrumentation for pelvic tumor treatment. Implant and template manufactured by Mobelife NV, Leuven, Belgium. **(A)** A pre-operative X-ray radiograph; **(B)** the implant; **(C)** the patient-specific guide; and **(D)** a post-operative X-ray radiograph (Courtesy of Prof. Dr. K Siebenrock, Inselspital, University of Bern, Switzerland).

Recently, computer-assisted surgery using individual templates has gained increasing attention. Initially developed for pedicle screw fixation (32), such a technique has been successfully reintroduced to the market for total knee arthroplasty (33, 68, 69), hip resurfacing (34, 70), total hip arthroplasty (35), and pelvic tumor resection (71, 72) (See Figure 7 for an example). It should be noted that most of the individual templates are produced using additive manufacturing techniques, while most of the associated implants are produced conventionally.

## 4 Future Perspectives

Despite its touted advantages, such as decreased radiation exposure to the patient and the surgical team for certain surgical procedures and increased accuracy in most situations, surgical navigation has yet to gain general acceptance among orthopedic surgeons. Although issues related to training, technical difficulty, and learning curve are commonly presumed to be major barriers to the acceptance of surgical navigation, a recent study (73) suggested that surgeons did not select them as major weaknesses. It has been indicated that barriers to adoption of surgical navigation are neither due to a difficult learning curve nor to a lack of training opportunities. The barriers to adoption of navigation are more intrinsic to the technology itself, including intra-operative glitches, unreliable accuracy, frustration with intra-operative registration, and line-of-sight issues. These findings suggest that significant improvements in the technology will be required to improve the adoption rate of surgical navigation. Addressing these issues from the following perspectives may provide solutions in the continuing effort to implement surgical navigation in everyday clinical practice.

**Figure 8:**
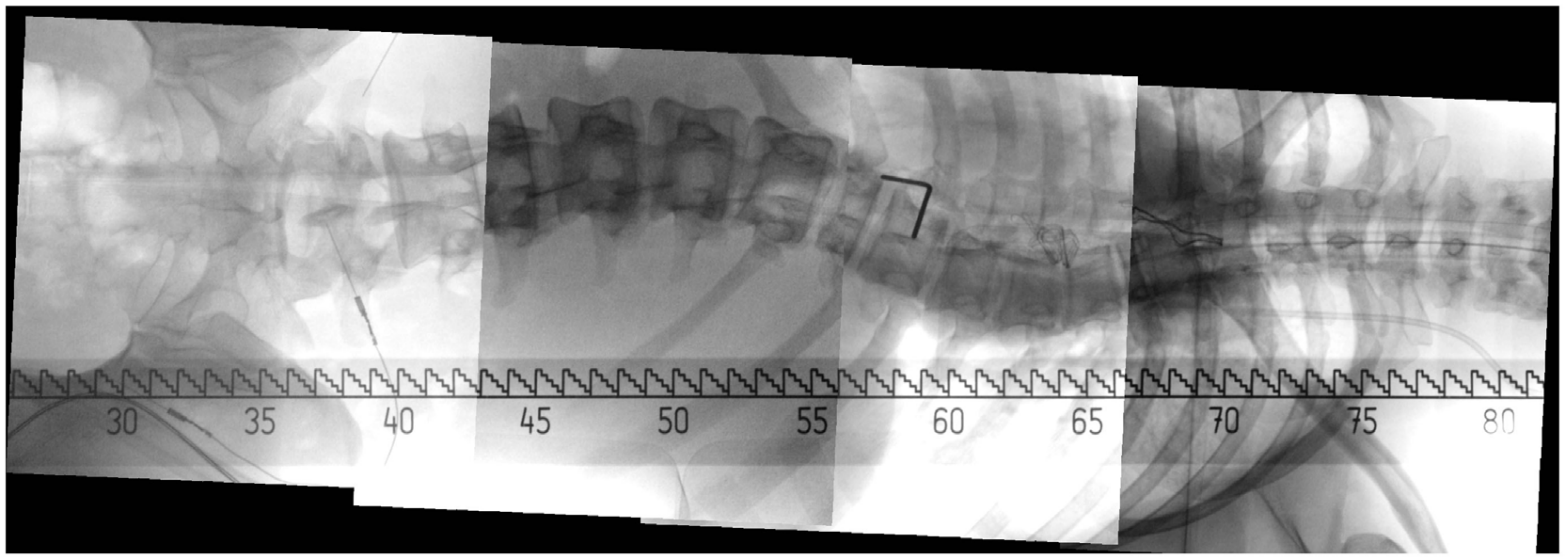
**Image stitching for spinal interventions**. Several small field-of-view C-arm images are stitched into one big image to depict the entire spine.

**Figure 9:**
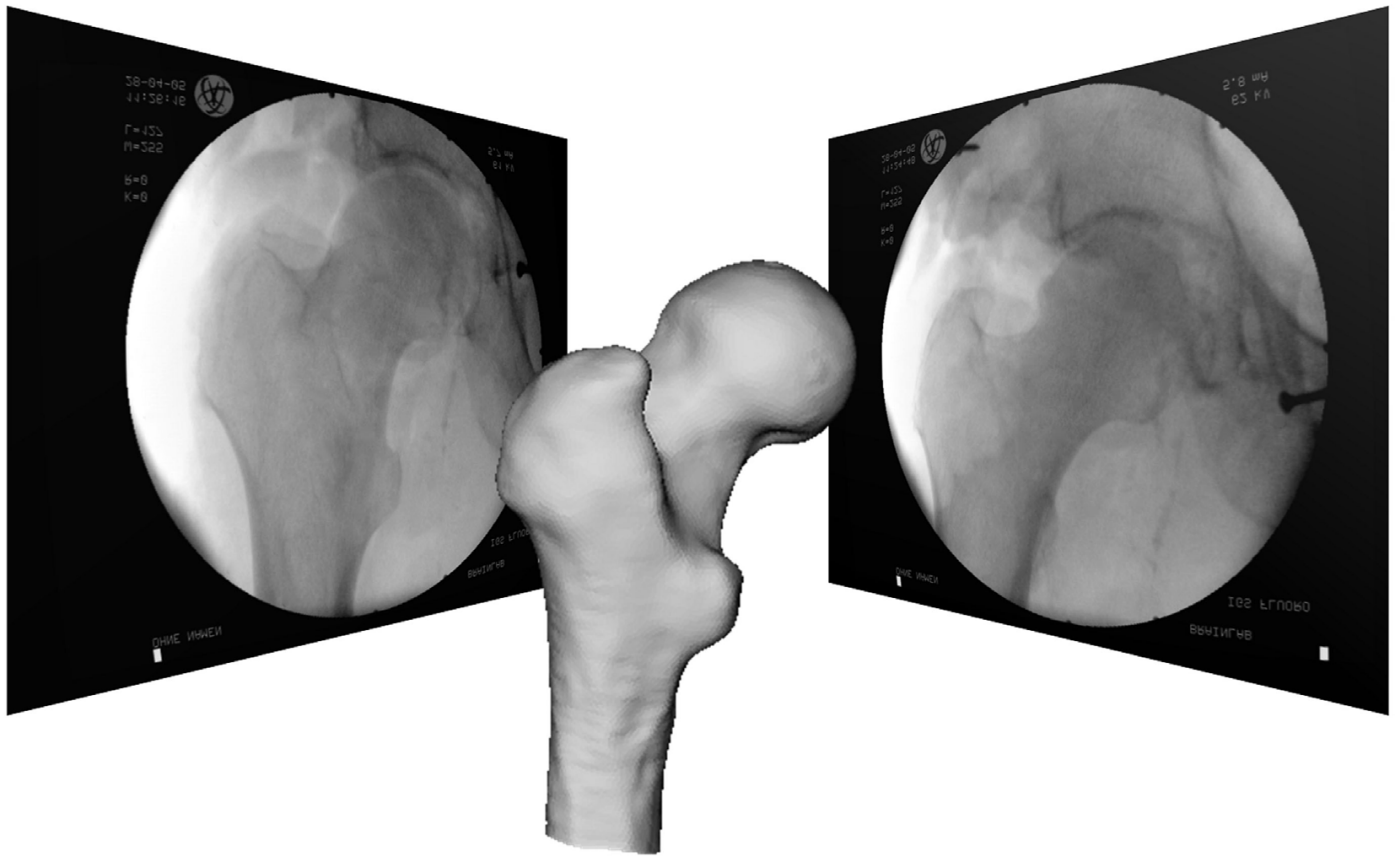
**Example of statistical shape model-based 2-D–3-D reconstruction**. Reconstruction of bone surface from two calibrated fluoroscopic images and a statistical shape model using deformable registration.

- *2-D or 3-D Image Stitching*. Long bone fracture reduction and spinal deformity correction are two typical clinical applications that frequently use the C-arm in its operation. Such a surgery usually involves corrective maneuvers to improve the sagittal or coronal profile. However, intra-operative estimation of the amount of correction is difficult, especially in longer instrumentation. Mostly, anteroposterior (AP) and lateral (LAT) fluoroscopic images are used but have the disadvantage to depict only a small portion of the target structure in a single C-arm image due to the limited field of view of a C-arm machine. As such, orthopedic surgeons nowadays are missing an effective tool to image the entire anatomical structure such as the spine or long bones during surgery for assessing the extent of correction. Although radiographs, obtained either by using a large field detector or by image stitching, can be used to image the entire structure, they are usually not available for intra-operative interventions. One alternative is to develop methods to stitch multiple intra-operatively acquired small fluoroscopic images to be able to display the entire structure at once (74, 75). Figure 8 shows an image stitching example for spinal intervention. The same idea can be extended to 3-D imaging to create a panoramic cone beam computed tomography (76). At this moment, fast and easy-to-use 2-D or 3-D image stitching systems are still under development and as the technology evolves, surgical benefits and improved clinical outcomes are expected.
- *Image Fusion*. Fusion of multimodality pre-operative image such as various MRI or CT datasets with intra-operative images would allow for visualization of critical structures such as nerve roots or vascular structures during surgical navigation. Different imaging modalities provide complementary information regarding both anatomy and physiology. The evidence supporting this complementarity has been gained over the last few years with increased interest in the development of platform hardware for multimodality imaging. Because multimodality images by definition contain information obtained using different imaging methods, they introduce new degrees of freedom, raising questions beyond those related to exploiting each single modality separately. Processing multimodality images is then all about enabling modalities to fully interact and inform each other. It is important to choose an analytical model that faithfully represents the link between the modalities without imposing phantom connections or suppressing existing ones. Hence, it is important to be as data driven as possible. In practice, this means making the fewest assumptions and using the simplest model, both within and across modalities. Example models include linear relationships between underlying latent variables, use of model-independent priors, such as sparsity, non-negativity, statistical independence, low-rank, and smoothness, or both. Such a principle has been successfully applied to solving challenging problems in a variety of applications (77, 78). Despite the evident potential benefit, the knowledge of how to actually exploit the additional diversity that multimodality images offer is currently at its preliminary stage and remains open for exploration.
- *Statistical Shape Modeling*. Statistical shape modeling has been shown to be useful for predicting 3-D anatomical shape and structures from sparse point sets that are acquired with the SDA technique. Such a technique is heavily employed in the so-called “image-free” navigation systems that are commercially available in the market, mainly for knee and hip surgery. However, with the availability of statistical shape models of other anatomical regions, the technique could be applied to any part of the skeleton. Such approaches bear significant potential for future development of computer navigation technology since they are not at all bound to the classical pointer-based acquisition of bony features. In principle, the reconstruction algorithms can be tuned to any type of patient-specific input, such as e.g., intra-operatively acquired fluoroscopic images (79) or tracked ultrasound (30), thereby potentially enabling new minimally invasive procedures. Figure 9 shows an example of bone surface reconstruction from calibrated fluoroscopic images and a statistical shape model. Moreover, prediction from statistical shape models is possible not only for the geometric shape of an object. Given statistical shape and intensity models, “synthetic CT scans” could be predicted from intra-operatively recorded data after a time-consuming computation (80). With more and more computations shifted from CPUs to graphics processing units (GPUs), it is expected that a whole statistical shape modeling-based techniques will be used in more and more CAOS systems.
- *Biomechanical Modeling*. Numerical models of human anatomical structures may help the surgeon during the planning, simulation, and intra-operative phases with the final goal to optimize the outcome of orthopedic surgical interventions. The terms “physical” or “biomechanical” are often used. While most of existing biomechanical models serve for the basic understanding of physical phenomena, only a few have been validated for the general prediction of consequences of surgical interventions.
  The situation for patient-specific models is even more complex. To be used in clinical practice, ideally the exact knowledge of the underlying geometrical tissue configuration and associated mechanical properties as well as the loading regime is required as input for appropriate mathematical frameworks. In addition, these models will not only be used pre-operatively, but need to function also in near real-time in the operating theater.
  First attempts have been made to incorporate biomechanical simulation and modeling into the surgical decision-making process for orthopedic interventions. For example, a large spectrum of medical devices exists for correcting deformities associated with spinal disorders. Driscoll et al. (81) developed a detailed volumetric finite element model of the spine to simulate surgical correction of spinal deformities and to assess, compare, and optimize spinal devices. Another example was presented in Ref. (82) where the authors showed that with biomechanical modeling the instrumentation configuration can be optimized based on clinical objectives. Murphy et al. (83) presented the development of a biomechanical guidance system (BGS) for PAO. The BGS aims to provide not only real-time feedback of the joint repositioning but also the simulated joint contact pressures.
  Another approach is the combined use of intra-operative sensing devices with simplified biomechanical models. Crottet et al. (84) introduced a device that intra-operatively measures knee joint forces and moments and evaluated its performance and surgical advantages on cadaveric specimens using a knee joint loading apparatus. Large variation among specimens reflected the difficulty of ligament release and the need for intra-operative force monitoring. A commercial version of such a device (e-LIBRA Dynamic Knee Balancing System – Synvasive Technology, El Dorado Hills, CA, USA) became available in recent years and is clinically used [see, e.g., Ref. (85)]. It is expected that incorporation of patient-specific biomechanical modeling into CAOS systems with or without the use of intra-operative sensing devices may eventually increase the quality of surgical outcomes. Research activities must focus on existing technology limitations and models of the musculoskeletal apparatus that are not only anatomically but also functionally correct and accurate.
- *Musculoskeletal Imaging*. Musculoskeletal imaging is defined as the imaging of bones, joints, and connected soft tissues with an extensive array of modalities, such as X-ray radiography, CT, ultrasonography, and MRI. For the past two decades, rapid but cumulative advances can be observed in this field, not only for improving diagnostic capabilities with the recent advancement on low-dose X-ray imaging, cartilage imaging, diffusion tensor imaging, MR arthrography, and high-resolution ultrasound, but also for enabling image-guided interventions with the introduction of real-time MRI or CT fluoroscopy, molecular imaging with PET/CT, and optical imaging into OR (86).
  One recent advancement that has found a lot of clinical applications is the EOS 2-D/3-D image system (EOS Imaging, Paris, France), which was introduced to the market in 2007. The EOS 2-D/3-D imaging system (87) is based on the Nobel prize-winning work of French physicist Georges Charpak on multiwire proportional chamber, which is placed between the X-rays emerging from the radiographed object and the distal detectors. Each of the emerging X-rays generates a secondary flow of photons within the chamber, which in turn stimulate the distal detectors that give rise to the digital image. This electronic avalanche effect explains why a low dose of primary X-ray beam is sufficient to generate a high-quality 2-D digital radiograph, making it possible to cover a field of view of 175 cm by 45 cm in a single acquisition of about 20-s duration (88). With an orthogonally co-linked, vertically movable slot-scanning X-ray tube/detector pairs, EOS has the benefit that it can take a pair of calibrated posteroanterior (PA) and LAT images simultaneously (89). EOS allows the acquisition of images while the patient is in an upright, weight-bearing (standing, seated, or squatting) position, and can image the full length of the body, removing the need for digital stitching/manual joining of multiple images (90). The quality and nature of the image generated by EOS system is comparable or even better than computed radiography (CR) and digital radiography (DR) but with much lower radiation dosage (89). It was reported by Illés et al. (89) that absorbed radiation dose by various organs during a full-body EOS 2-D/3-D examination required to perform a surface 3-D reconstruction was 800–1000 times less than the amount of radiation during a typical CT scan required for a volumetric 3-D reconstruction. When compared with conventional or digitalized radiographs (91), EOS system allows a reduction of the X-ray dose of an order 80–90%. The unique feature of simultaneously capturing a pair of calibrated PA and LAT images of the patient allows a full 3-D reconstruction of the subject’s skeleton (89, 92, 93). This in turn provides over 100 clinical parameters for pre- and post-operative surgical planning (89). With a phantom study, Glaser et al. (94) assessed the accuracy of EOS 3-D reconstruction by comparing it with 3-D CT. They reported a mean shape reconstruction accuracy of 1.1 ± 0.2 mm (maximum 4.7 mm) with 95% confidence interval of 1.7 mm. They also found that there was no significant difference in each of their analyzed parameters (*p* > 0.05) when the phantom was placed in different orientations in the EOS machine. The reconstruction of 3-D bone models allows analysis of subject-specific morphology in a weight-bearing situation for different applications to a level of accuracy that was not previously possible. For example, Lazennec et al. (95) used the EOS system to measure pelvis and acetabular component orientations in sitting and standing positions. Further applications of EOS system in planning total hip arthroplasty include accurate evaluation of femoral offset (96) and rotational alignment (97). The low dose and biplanar information of the EOS 2-D/3-D imaging system introduce key benefits in contemporary radiology and opens numerous and important perspectives in CAOS research.

## 5 Conclusion

About two decades have passed since the first robot and navigation systems for CAOS were introduced. Today, this technology has emerged from the laboratory and is being routinely used in the operating theater and might be about to become state-of-the-art for certain orthopedic procedures.

Still we are at the beginning of a rapid process of evolution. Existing techniques are being systematically optimized and new techniques will constantly be integrated into existing systems. Hybrid CAOS systems are under development, which will allow the surgeon to use any combinations of the above-described concepts to establish virtual object information. New generations of mobile imaging systems, inherently registered will soon be available. However, research focus should particularly be on alternative tracking technologies, which remove drawbacks of the currently available optical tracking and magnetic devices. This in turn will stimulate the development of less or even non-invasive registration methods and referencing tools. Force sensing devices and real-time computational models may allow establishing a new generation of CAOS systems by going beyond pure kinematic control of the surgical actions. For key-hole procedures, there is distinct need for smart EEs to complement the surgeon in its ability to perform a surgical action.

All these new techniques and devices need to be carefully evaluated first in the laboratory setting and then clinically. However, it may be hypothesized that the ultimate acceptance of robotic or navigated orthopedic surgery will be contributed to the proof of better long-term outcome of the respective intervention. Furthermore, this will serve as a basis for reimbursement, a prerequisite for successful market penetration. Consequently, more prospective and retrospective studies comparing the outcome of CAOS vs. non-CAOS procedures with long follow-up time such as the one reported in Ref. (98) will have to be conducted.

## Conflict of Interest Statement

The authors declare that the research was conducted in the absence of any commercial or financial relationships that could be construed as a potential conflict of interest.
